# Under the Umbrella of Clinical Pharmacology: Inflammatory Bowel Disease, Infliximab and Adalimumab, and a Bridge to an Era of Biosimilars

**DOI:** 10.3390/pharmaceutics14091766

**Published:** 2022-08-24

**Authors:** Zvonimir Petric, Joao Goncalves, Paulo Paixao

**Affiliations:** 1 Department of Pharmacological Sciences, Research Institute for Medicines (iMed.ULisboa), Faculty of Pharmacy, University of Lisbon, 1649-004 Lisboa, Portugal; ppaixao@ff.ul.pt; 2 Biopharmaceutical and Molecular Biotechnology Unit, Research Institute for Medicines (iMed.ULisboa), Faculty of Pharmacy, University of Lisbon, 1649-004 Lisboa, Portugal; joao.goncalves@ff.ul.pt

**Keywords:** monoclonal antibodies, inflammatory bowel disease, anti-TNF-α agents, infliximab (IFX), adalimumab (ADL), immunogenicity, effectiveness, safety, biosimilars

## Abstract

Monoclonal antibodies (MAbs) have revolutionized the treatment of many chronic inflammatory diseases, including inflammatory bowel disease (IBD). IBD is a term that comprises two quite similar, yet distinctive, disorders—Crohn’s disease (CD) and ulcerative colitis (UC). Two blockbuster MAbs, infliximab (IFX) and adalimumab (ADL), transformed the pharmacological approach of treating CD and UC. However, due to the complex interplay of pharmacology and immunology, MAbs face challenges related to their immunogenicity, effectiveness, and safety. To ease the burden of IBD and other severe diseases, biosimilars have emerged as a cost-effective alternative to an originator product. According to the current knowledge, biosimilars of IFX and ADL in IBD patients are shown to be as safe and effective as their originators. The future of biosimilars, in general, is promising due to the potential of making the health care system more sustainable. However, their use is accompanied by misconceptions regarding their effectiveness and safety, as well as by controversy regarding their interchangeability. Hence, until a scientific consensus is achieved, scientific data on the long-term effectiveness and safety of biosimilars are needed.

## 1. Introduction

According to the definition, biologic therapy (biologic therapeutic) is a medicine that is made from living organisms (or its products), and is used for the treatment of diseases, as well as for disease prevention or diagnosis [1]. The definitions given by the regulatory agencies in the EU, the European Medicines Agency (EMA), and in the USA, the Food and Drug Administration (FDA), are more or less similar [2,3]. In 2020, the FDA rephrased its definition by adding that biological product actually refers to all proteins, including any alpha amino acid polymer greater than 40 amino acids [3]. This change, however, has regulatory repercussions without being generally relevant for the clinical practice.

Biopharmaceutical innovation and the implementation of biologic therapy have revolutionized the treatment options for many diseases, from the field of oncology to chronic and inflammatory autoimmune disorders, such as inflammatory bowel disease (IBD) and rheumatoid arthritis (RA), where prior pharmacological attempts with conventional therapy have often been unsuccessful. Hence, it is no surprise that, in the eyes of patients and clinicians, biologic therapeutics are perceived as a game-changing therapeutic modality.

Biologic therapeutics (also called biologics, biologic medicines, biological products, biologics-based medicines, biotherapeutics, biopharmaceuticals, etc.) are well-known as relatively complex molecules produced via a highly sophisticated biotechnological methodology; hence, their high price is unsurprising. Biologics are the fastest-growing therapeutic modality. In 2020, the biologics market was valued to be around EUR 278 billion, while it is expected to reach an astonishing EUR 465 by 2026, according to [4]. Moreover, among the best-selling drugs in 2020, the top-selling was adalimumab (Humira^®^). Among the top ten drugs, six were monoclonal antibodies (MAbs) [5]. As the global market for biologics is obviously rising, it can be expected that such fast expansion in the coming years will pose big challenges for manufacturers and their production plans if they want to stay at the top in the ever-changing pharmaceutical landscape.

One of the most successful biologics is monoclonal antibodies (MAbs), also referred to as therapeutic antibodies in the case of their multi-indication use. The unique attribute of MAbs is their monospecificity, meaning that they recognize one particular antigenic determinant, i.e., an epitope, on a given molecule. Moreover, as antibodies are secreted by an individual hybridoma, they are completely identical immunoglobulin molecules, which show identical affinity to a target of medical interest, as well as identical physiochemical properties [6].

**Figure 1:**
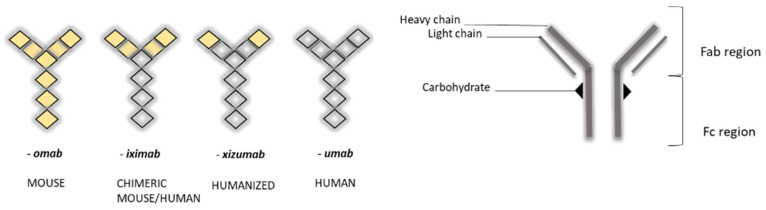
Schematic view of nomenclature for monoclonal antibodies (MAbs) (**left**) and general representation of their “Y” structure (**right**) [6]. Note the change in color, representing the differing humanization of antibodies.

The nomenclature of MAbs was devised by the World Health Organization (WHO), and follows the International Nonproprietary Nomenclature (INN) (Figure 1), except in the case of muromonab (murine monoclonal antibody) [7].

In the biopharmaceutical and pharmacological sense, biologics greatly differ from conventional therapy, also known as small-molecule drugs (Table 1). The fundamental differences between these therapeutic modalities, such as their size, chemical structure, physicochemical and biophysical properties, stability, complexity, and specificity, determine the differences in the processes of absorption, distribution, metabolism, and elimination (acronym ADME), i.e., pharmacokinetics (PK), as well as pharmacodynamics (PD). In addition, these differences also influence the way that both therapeutic modalities are manufactured [8].

## 2. General Concepts of Pharmacokinetics (PK) and Pharmacodynamics (PD) Related to MAbs

MAbs are 150 kDa immunoglobulin G (IgG) monoclonal antibodies, composed of two heavy chains and two light chains, which are linked by disulfide bonds, and which join to form a molecule resembling the letter “Y” (Figure 1). Tips of the “Y” (i.e., heavy + light chains) are called the variable region, while the stem portion of the “Y” (heavy + heavy chains) is called the constant region. Variable regions comprise an antigen-binding fragment (Fab), while constant regions comprise a fragment crystallizable (Fc) region. The Fab region binds to receptors on the cell’s surface, such as Fcγ receptors (FcγR) and neonatal Fc receptors (FcRn) [10,11].

The general pharmacological function of MAbs, for example, antagonism against tumor necrosis factor-alpha (TNF-α) cytokines, is dependent on the selective binding of the antibody to the target of interest (antigen) through variable regions. In addition to determining the antibody specificity of an antigen, variable regions also determine the potency of MAbs. On the other hand, the constant region impacts the functional effects of MAbs, such as its developability (i.e., biophysical properties), immunogenicity (i.e., ability to provoke an immune reaction), and effector functions (i.e., binding to receptors and PD). MAbs can also have post-translational modifications, such as amino acid and carbohydrate (glycosylation) modifications. Even a slight change in the constant (or variable) region can have a big and unpredictable impact on the clinical pharmacology of MAbs, meaning that both PK (ADME) and PD (efficacy, effectiveness, and safety) can be altered [10,11].

As proteins, MAbs have a very low oral bioavailability, poor gastrointestinal stability, and poor lipophilicity, which makes them unsuitable for oral administration. Their apparent volume of distribution is considered to be relatively small and often limited to the circulatory tissue. In a steady state, typical values of the apparent volume of distribution (Vd) are within the range of 3.5–7 L, which indicates the limited distribution of MAbs to vascular and interstitial spaces [9].

The transfer of MAbs from plasma to interstitial space depends on the convective transport (as opposed to diffusion seen with small-molecule drugs), while the rate is determined by capillary permeability. Convection depends on the hydrostatic and osmotic pressure gradients between blood and tissue, but also on the vascular endothelium containing pores, which differ in amount and size. Some tissues may have a more “leaky” endothelium, while capillaries in the brain and their endothelial cells are actually impermeable, meaning that concentrations of MAbs in the brain are less than 1% relative to plasma concentrations [12].

It is also important to mention that MAbs administered via extravascular routes, i.e., intramuscularly (i.m.) or subcutaneously (s.c.), will have a rate of absorption dependent on the convective transport and lymph flow [13,14]. While the lymph volume can influence the apparent volume of distribution in a steady state [14], it can also be stated that the distribution of MAbs is relatively fast, while elimination (by either excretion or catabolism) is relatively slow [9,13].

Due to their large size, MAbs are not eliminated by the kidneys in normal situations, while biliary excretion is also not considered to be relevant as the number of MAbs eliminated in this way is very small. Hence, the main elimination of MAbs is facilitated by proteolytic catabolism. Catabolism is mediated via lysosomal degradation (to amino acids) after the uptake of the antibody into cells by two mechanisms. The first uptake mechanism is pinocytosis, a form of unspecific fluid-phase endocytosis, which takes place on the vascular monolayer of endothelial cells. Pinocytosis is not limited to any particular organ or tissue, but instead occurs throughout the body where rich capillary beds are located, i.e., endothelial cells (liver, muscle tissue, skin, gastrointestinal tract, etc.) [12,13,15]. The second uptake mechanism leading to MAb elimination is receptor-mediated endocytosis, where an MAb’s Fc domain interacts with Fc cell receptors (FcγR), leading to endocytotic internalization, and the subsequent inactivation of MAbs (via lysosomal degradation). Various types of immune cells, such as monocytes, macrophages, natural killer (NK) cells, and dendritic cells, express FcγR on their surface membrane [12,16]. However, one additional interaction is related to receptor-mediated endocytosis, which implies the Fab-binding domain of the antibody to its specific target, i.e., epitope. This is known as a specific clearance pathway of MAbs, and is often referred to as target-mediated drug disposition (TMDD) [9,13,17].

TMDD is considered to be a PK, i.e., drug distribution, phenomenon. It has a lower elimination capacity compared to unspecific pinocytosis and, thus, can be saturable (contrary to an unspecific pinocytosis clearance mechanism that typically shows a linear behavior within the approved therapeutic dosage range). An in-depth explanation of TMDD is beyond the scope of this review; however, we only present its general relevance. In short, TMDD occurs due to a very high affinity and very high binding specificity of the drug for its relatively low-capacity (i.e., low-density) pharmacological target. This phenomenon can be viewed as an example of how PD impacts PK (usually PK impacts PD), and as such, it is relevant to the disposition of biologics, contrary to common belief, as well as for some small-molecule drugs. TMDD could lead to the increased elimination of a drug due to the fact that the drug–target complex molecules can become endocytosed and degraded [13,18,19]. Hence, drugs cleared primarily via TMDD will show dose-dependent nonlinear elimination (even at therapeutic concentrations for some drugs), so TMDD can be considered as an important contributing factor for drug elimination. However, due to the generally high therapeutic concentrations of MAbs used in the clinical setting, TMDD will not usually be the main factor that contributes to increased drug clearance, as there will be the sufficient fraction of a drug (unbound), compared to the fraction (bound, i.e., captured) on the target (receptor). As an example, antibodies against soluble antigens, e.g., tumor necrosis factor-alpha: TNF-α, infliximab (IFX), and adalimumab (ADL), are administered in a high dose and display linear elimination within that therapeutic range [9,13]. In conclusion, the rate of drug elimination mediated through TMDD will mainly depend on the drug dose, target capacity (density), drug affinity, binding specificity, and the rate of catabolism [13].

Other molecular aspects of pharmacology, which add an additional layer of complexity to the PKPD properties of MAbs, are target turnover rate, changes in the patterns of glycosylation, off-target binding, immunogenicity (i.e., generation of anti-drug antibodies: ADAs) and the FcRn-mediated recycling of MAbs. Due to immunogenicity, i.e., antibody–ADA immune complexes, we can also expect changes in antibody disposition, such as increased clearance and reduced half-life [9]. On the other hand, FcRn-mediated recycling serves as a salvage pathway for MAbs, as it protects antibodies from lysosomal degradation and, thus, it partially counteracts the clearance process. Despite being a capacity-limited process (such as TMDD), FcRn-mediated recycling has a very important PKPD consequence, which is the prolongation of elimination half-life and, consequently, a longer duration of pharmacological effects [20]. Hence, FcRn-mediated recycling can be exploited as a prospective tool for improving the pharmacological properties of antibodies. On the other hand, blocking the FcRn activity was shown to be a good strategy for the treatment of myasthenia gravis. Currently, nipocalimab (anti-FcRn monoclonal antibody) is under clinical trials in adults (phase III) and children (phase II) [21,22]. Similarly, efgartigimod alfa (antibody Fc fragment) is currently expected to be approved in the EU for the treatment of generalized myasthenia gravis [23]. It is worth mentioning how the expression of Fc receptors in different pathologies can result in a variety of immunological responses, e.g., autoimmunity, inflammation, or allergies. Additionally, the therapeutic effectiveness of MAbs is found to be related to the genetic variants of Fc receptors in individuals [24]. This also means that in IBD, the dysregulation of FcR signaling [25] could have a positive (or negative) influence on the clinical response. In order to be “druggable” enough, the MAb drug target should be easily available and tissue-specific, while, at the same time, maintaining a low receptor turnover rate and low density. The latter properties offer less frequent dosing, or using a drug in lower amounts [26]. In addition to the previously described molecular PKPD complexities related to MAbs, there are also patient-related complexities, which cause interindividual variability in PK, in turn affecting PD. These differences are mostly related to age, pharmacogenetic profile (genetic polymorphisms), concomitant medications, immunogenicity (ADAs), and disease/health status [9,14]. Hence, as the knowledge on PKPD, inter-patient variability, and underlying pathology is still limited, it is important to bear in mind their joint influence on the pharmacological success of MAbs [14,17]. Therefore, clinicians often use biomarkers and clinical endpoints as a surrogate for pharmacological success. For example, in the case of IBD, the serum level of C-reactive protein (CRP), or a fecal calprotectin, and the status of mucosal healing are of great help in monitoring disease progression and evaluating the success of pharmacological intervention [9,27,28,29].

## 3. Inflammatory Bowel Disease (IBD)

**Figure 2:**
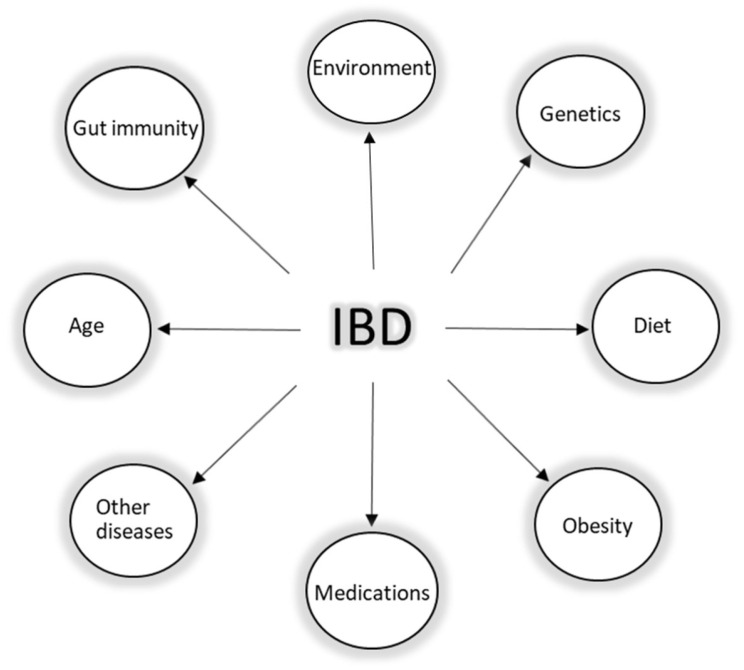
Factors influencing the etiology and pathophysiology of IBD [30,32,34].

IBD is an umbrella term, which is mainly used to describe a group of contrasting yet related intestinal disorders: Crohn’s disease (CD) and ulcerative colitis (UC). Both disorders are characterized by non-infectious chronic relapsing episodes of inflammation of the gastrointestinal tract, probably caused by a dysregulation of immune response to the gut microbiome in genetically susceptible individuals [30,31]. As the etiology and pathophysiology of IBD is puzzling (Figure 2), so far, it has been established that genetic risk factors, environmental factors, lifestyle, mucosal immunity via the intestinal barrier, and the gut microbiome (intestinal dysbiosis) all play a role in the development of the disease. Despite the knowledge on the interplay of these factors, the health burden of IBD is still globally rising. In 2017, according to sources, the number of cases worldwide was 6.8 million, and currently, around 7 million people are living with IBD worldwide [32,33]. Additionally, the impact of such a burden on the health system in the next few years, especially if we consider the global trends in aging of the population, may become cumbersome.

The mortality from IBD can be considered to be relatively low, and the age of patients at the time of diagnosis is often relatively young, but at the same time, highly industrialized countries have a greater IBD burden when compared to countries in transition [35,36,37]. However, it is not yet certain if the countries in transition, when the prevalence of IBD is much higher than it is now, will be able to offer biologics to all patients. Instead, the solution may lie in biosimilars, which could be a viable cost-effective alternative to ease the health–economic burden [37].

Crohn’s disease (CD) can occur anywhere in the gastrointestinal tract (GI), and the inflammation is transmural, i.e., all layers of the bowel may be affected. CD can be classified according to the disease location (terminal ileal—L1; colonic—L2; ileocolic—L3; or isolated upper GI—L4), or according to behavior (non-stricturing and non-penetrating—B1; stricturing—B2; penetrating—B3). Disease localization influences the presentation of the disease, but generally speaking, patients with CD suffer from diarrhea, often feel abdominal discomfort and pain, and experience substantial weight loss. If the disease affects the small bowel, it can result in the malabsorption of iron, cobalamin (vitamin B12), and bile acids. Rectal bleeding is not very common, except in the case of disease localization in that area. CD in the upper gastrointestinal tract can be manifested by aphthous ulcers, vomiting, and nausea. If CD is left untreated, most patients will develop complications of CD, such as perirectal abscesses, and anorectal and anal fistulas. Serious complications are also abdominal abscesses and colorectal cancer [38]. It is worth stressing how common it is that diarrheas lead to the loss of potassium, magnesium, and other electrolytes (as well as various vitamins), which has a negative effect on many physiological processes, such as cardiac rhythm, gastric motility, and renal function. The clinical symptoms of such a scenario include muscle weakness, arrhythmias, increased insulin resistance, tremor, encephalopathy, and bone disorders [39].

Ulcerative colitis (UC), on the other hand, is characterized by continuous mucosal inflammation (with no patchiness), which starts in the rectum and can be extended to the rest of the large intestine, except for the small bowel. UC has various classifications such as the Montreal consensus (based on anatomical regions): ulcerative proctitis—E1; distal or left-sided UC—E2; and extensive UC—E3. The severity of the disease (or disease activity index: DAI) is classified by the Mayo Score based on four parameters (stool pattern, rectal bleeding, endoscopic findings, and the physician’s assessment). UC is also associated with colorectal cancer, while a rare but potentially fatal complication of UC is toxic megacolon [38].

Extraintestinal manifestations of IBD (summarized in Table 2) can affect other parts of the body such as the musculoskeletal, vascular, hepato-biliary, metabolic, renal, pulmonary, ocular and oral systems, as well as the skin. Patients may often develop mental health problems and have difficulties with body image and sexuality [40,41].

When looking for the indicators of an active IBD, clinicians are interested in blood, fecal, and serological markers. The monitoring of disease activity in IBD is absolutely necessary as it influences the choice of pharmacological treatment for an individual patient. The most common blood indicators of active IBD include C-reactive protein (CRP) and erythrocyte sedimentation rate (ESR) [42]. CRP, as a biomarker of acute inflammation, is a protein synthesized in the liver as an answer to proinflammatory cytokines [43]. If CRP levels are, in general, below 10 mg/L, it indicates the remission stage of IBD and correlates with a decrease in endoscopic disease activity in IBD patients [44,45,46]. Hence, CRP can be viewed as an indicator of disease activity, as well as a surrogate for predicting clinical response [44]. Other helpful tools for IBD diagnosis are stool specimen analysis for lactoferrin and calprotectin, as well as serological markers. For example, perinuclear anti-neutrophil cytoplasmic antibodies (pANCAa) are used to differentiate CD from UC, anti-saccharomyces cerevisiae antibodies (ASCAa), or antibodies against exocrine pancreas (PABs), etc. Lastly, some researchers question the general sensitivity and specificity of commonly used biomarkers such as CRP, so new strategies and novel biomarkers are still being explored. The most recent studies suggest oncostatin M (OSM) and serum miRNAs as novel biomarkers for the monitoring of IBD [47,48,49].

## 4. Short Immunological Background of IBD

**Figure 3:**
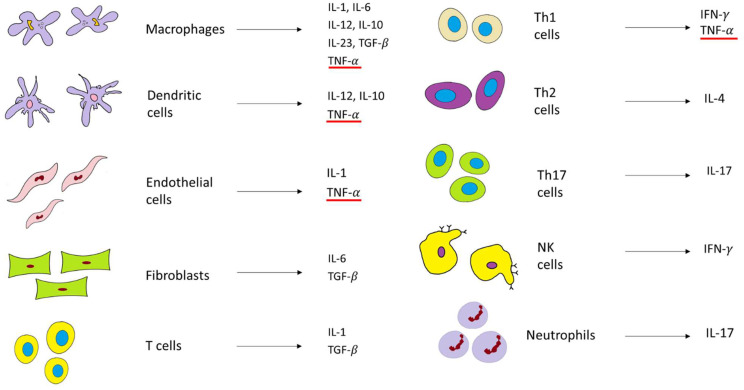
Main cells and cytokines involved in the immune response in IBD. The red line depicts TNF-α as the main proinflammatory cytokine of the inflammatory cascade [50].

In the healthy gut, Toll-like receptors (TLRs), as pathogen-sensitive innate immune receptors found on monocytes, macrophages, dendritic cells, and epithelial cells, help to maintain the intestinal epithelial barrier. This protective mechanism involves nuclear factor kappa-light-chain-enhancer of activated B cells (NF-κB), which triggers the expression of inflammatory molecules such as TNF-𝛼 and other chemokines. However, in patients with IBD, as barrier function is impaired, TLR signaling is hyperactivated and, consequently, the expression of TNF-α and IL-1, IL-2, IL-6, and IL-12 is elevated [34,50]. Currently, it is well-established that for the development of IBD, both innate and adaptive (or acquired) immune responses need to be engaged (Figure 3). The innate immune response includes the same cells in CD and UC. Hence, IBD studies show similar increases in macrophages and dendritic cells with the increase in pro-inflammatory cytokines such as TNF-𝛼, a key player in IBD, and others, such as interleukin 1 (IL1), IL-12, and IL-6. On the contrary, the adaptive immune response has a completely different pathway in CD, compared to UC. The inflammation in CD is mediated via the T helper type 1 and T helper type 17 cell-mediated cytokine profile (Th1 and Th17). The inflammation in UC is mediated via natural killer T cells (NK cells) and T helper type 2 cell-mediated cytokine profile (Th2) (Figure 3) [34,50].

## 5. Pharmacological Armamentarium of IBD: Targeting TNF-α with Anti-TNF-α Agents—IFX and ADL

Some of the main proinflammatory cytokines include TNF-α, IL-1, and IL-6 (Figure 3). TNF-α is considered to be at the top of the inflammatory cascade and acts as a key player in IBD pathogenesis [50]. In healthy (physiological) conditions, as previously stated, TNF-α is a beneficial immune mediator that is responsible for maintaining balanced gut immune homeostasis. However, in the inflammatory state, TNF-α is produced relatively quickly (within one hour) compared to other proinflammatory cytokines. Moreover, TNF-𝛼 has a high potency, as it binds to the receptors with a very high affinity [51,52]. As it is a transmembrane protein (tm) and expressed on the cell surface, tmTNF-α (also known as mTNF-α) is cleaved by a metalloproteinase, which liberates another form of TNF known as soluble TNF-α (sTNF-α). sTNF-α can be found (and measured) as a homotrimer circulating in the blood. Both mTNF-α and sTNF-α are bound to transmembrane receptor molecules p55/p60 (also known as TNFR1) and p75/p80 (also known as TNFR2), which can also exist in their soluble forms. mTNF-α is a ligand for both these receptors, and their overexpression is additionally upregulated by interferons [53]. 

The binding of TNF-α to receptors forms TNF–TNFR complexes and leads to the overexpression of inflammatory cytokines, cell apoptosis, and necrosis, or alternatively, cell survival, depending on the signaling cascade. One interesting phenomenon related to TNF-α is the possibility of autoupregulation and the creation of a positive pro-inflammatory feedback loop, which further amplifies the inflammatory process [54]. Therefore, the concept of the pharmacological targeting of this pleiotropic cytokine [55] was a revolutionary step in the early 1990s, when the first experiments confirmed the proof of concept [56,57].

**Figure 4:**
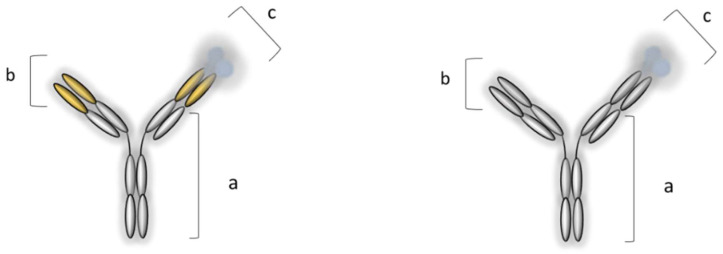
Monoclonal IgG1 anti-TNF-α antibodies (MAbs): infliximab, IFX (on the **left**), and adalimumab, ADL (on the **right**). **IFX**: (a) Human IgG1 constant region, (b) mouse antigen-binding variable region, and (c) homotrimer of TNF-α; **ADL**: (a) human IgG1 constant region, (b) human antigen-binding variable region, and (c) homotrimer of TNF-α [6,60].

A few years later, the pharmacological armamentarium of IBD, in addition to conventional therapy, was supplemented by IFX, approved for medical use by the FDA in 1998, while the approval of ADL followed four years later. IFX is a chimeric (human–murine) monoclonal IgG1 anti-TNF-α antibody, while ADL is a fully human monoclonal IgG1 anti-TNF-α antibody (Figure 4) [58,59].

**Figure 5:**
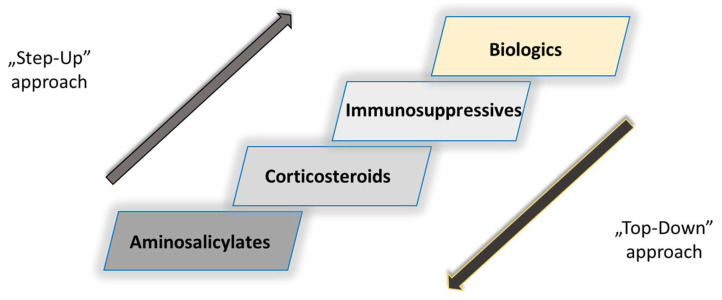
Pharmacological armamentarium of IBD and paradigm shift in the management of IBD [61].

Both anti-TNF-α agents revolutionized the treatment of IBD and contributed to a paradigm shift in the pharmacological management of IBD ( Figure 5 ).

The conventional treatment approach also known as “step-up” was replaced with the “top-down” approach (Figure 5). In other words, this is the concept of gradually introducing different pharmacological drug classes in the case of IBD progression, starting first with aminosalicylates (5-aminosalicylic acid and sulfasalazine), corticosteroids (prednisone) and immunosuppressives (azathioprine, 6-mercaptopurine), and as the last option, biologics (IFX and ADL), which eventually became first-choice drugs [61].

Therapeutic goals also shifted as clinical remission changed from being based on disease symptomatology only to an objective criterion, such as endoscopic mucosal healing, i.e., the regression and disappearance of endoscopic lesions, which is known as endoscopic remission [62]. Such a new approach of IBD treatment is named “treat-to-target approach” [63]. Its proposed benefits are reducing the disease burden at early stages of IBD and improving clinical outcome. However, in this approach, MAbs should be applied tentatively, as some researchers suggest, because not all IBD patients will require immediate treatment with biologics as the first-line therapy. On the other hand, a 2-year open-label randomized EU trial [64] showed that even an early introduction of more potent treatments in CD (e.g., infliximab with azathioprine) resulted in a better outcome.

**IFX** (≈149 kDa; pharmacotherapeutic group—immunosuppressants; anatomical therapeutic chemical (ATC) code L04AB02) was introduced in Europe 23 years ago (Europe in 1999; USA in 1998) and was first approved for the treatment of CD (and later for UC). It was later approved for the treatment of conditions such as rheumatoid arthritis, ankylosing spondylitis, psoriatic arthritis, and psoriasis (a full list is shown in Table 3) [65]. Interestingly, the first clinical use of IFX was actually in a pediatric patient (12-year-old girl), whose symptoms of CD had not been relieved by conventional therapy at that time (prednisone, mesalazine, azathioprine, metronidazole, and enemas with salicylic acid). Initially, colonoscopy and tissue biopsy revealed severe inflammation and multiple aphthous lesions of the colon, as well as crypt abscesses with granuloma. Finally, after 2 years of discomfort, the patient received IFX, and immediately after the first dose, an improvement in her clinical symptoms was noticed [66].

Pharmacological studies showed that IFX binds and neutralizes both mTNF-α, expressed on immune cells (macrophages, T cells, dendritic cells, etc.) and sTNF-α, which in turn potentiates cell lysis via processes of antibody-dependent cellular cytotoxicity (ADCC), reverse signaling, and apoptosis [65]. In addition to ADCC, it is believed that IFX has one additional mechanism of action: complement-dependent cytotoxicity (CDC) [67]. However, in studies with peripheral blood mononuclear cells, IFX was not able to induce CDC [68]. Once the TNF-α is antagonized by IFX, effects that follow include the downregulation of proinflammatory cytokines, the reduced migration of immune cells (such as macrophages and T lymphocytes), and overall, a reduction in previously exaggerated immune response [58].

In some of the first clinical studies for CD [69], IFX showed a better clinical response compared with the placebo (41% vs. 12%, *p* < 0.008). Clinical remission was achieved in 33% of patients compared to the placebo (33% vs. 17%, *p* < 0.005), while 65% of patients had a primary endpoint reduction in the CDAI score (Crohn’s Disease Activity Index) of 70 points, compared to 17% who received the placebo (*p* < 0.001) [69]. The other two big studies of IFX in CD, namely, the ACCENT I [70] and SONIC trial [71], undoubtedly confirmed the superiority of IFX in terms of clinical response and remission and, as such, paved the way for IFX dosing in CD as we know it today. Therapy with IFX was shown to improve mucosal healing as a secondary endpoint in the SONIC trial measured on the CDEIS (Crohn’s Disease Endoscopic Index of Severity) scale [71].

In the ACT I and ACT II trials [72] in patients with UC, IFX was confirmed to be superior for treating the symptoms of disease, compared with the placebo. Clinical remission and mucosal healing were higher in the IFX group, and additional follow-up studies showed that IFX was able to sustain its effectiveness [72]. IFX was also found to improve the healing of perianal fistulas, interestingly, via local administration into inflamed tissue [73].

Regarding the pharmacokinetics, IFX administered via intravenous infusion (i.v.) shows a low apparent volume of distribution, with a long elimination half-life (Table 4). The area under the plasma concentration–time curve (AUC) increases proportionally with the dose of IFX, which indicates linear pharmacokinetics for the studied dose [58,74,75,76]. Additionally, IFX during repeated infusions (10 mg/kg, Q8W) in Crohn’s patients did not show signs of accumulation [77].

**ADL **(≈148 kDa; pharmacotherapeutic group—immunosuppressants; anatomical therapeutic chemical (ATC) code L04AB04) is the first fully human monoclonal IgG1 anti-TNF-α antibody to be developed, and it was first introduced in the USA in 2002 (Europe in 2003) [78]. Initially, the FDA approved the drug for the treatment of moderate to severe rheumatoid arthritis. In 2007, ADL received approval for the treatment of CD, and later for UC. Some additional indications include juvenile idiopathic arthritis and uveitis (a full list is shown in Table 3) [78].

ADL binds and neutralizes both forms of TNF-α with high affinity, and shows a high similarity to IFX in terms of binding kinetic characteristics and general descriptive pharmacodynamic effects. Remaining drugs from classes of anti-TNF-α agents (etanercept, certolizumab, and golimumab) show different binding characteristics, which could explain why these drugs, despite being from the same class, exhibit different levels of effectiveness across indications [79].

In the CLASSIC I and CLASSIC II trials, ADL induced and maintained clinical remission in patients with CD [80]. Moreover, patients on ADL were up to two times more likely to maintain remission at week 56, compared to the placebo. The CHARM trial [81] confirmed the effectiveness of ADL in the maintenance of clinical remission in patients with CD (40% vs. 17% for placebo group, *p* < 0.001), and the better healing of fistulas (33% vs. 13% for placebo group, *p* < 0.016). The EXTEND trial [82] confirmed overall superiority based on the mucosal healing rate of patients with CD (24% vs. 0% for placebo group, *p* < 0.001). The ACCES trial [83] showed that the occurrence of fistula healing in CD was greater in anti-TNF-α-naïve patients treated with ADL compared to those treated previously with IFX (60% vs. 28% for IFX group, *p* < 0.01). ADL was also shown to induce and sustain corticosteroid-free remission in both groups. In the CHOICE trial [84], ADL was shown to be effective in patients with CD who were primary non-responders to IFX (besides being an effective first-line therapy for anti-TNF-naïve patients).

In the ULTRA I and ULTRA II trials [85], the effectiveness of ADL was evaluated in UC. Results showed that ADL was also superior to the placebo in the induction of remission, clinical remission response, and mucosal healing. In addition, in the ULTRA II trial, approximately 40% of patients had prior exposure to the anti-TNF-α agent, meaning that ADL is beneficial to both primary non-responders and those who initially had a response that was not sustained [85].

Results from comparison studies of IFX vs. ADL in UC suggested that IFX is more effective in the induction of remission, response, and mucosal healing at week 8, while at week 52, both drugs are equally effective as a maintenance therapy [86]. However, in a very recent publication from Lee et al. [87], in a first head-to-head comparison in UC patients, results suggested that both drugs have comparable remission rates at week 8 (47% vs. 56.7%, *p* = 0.364) and week 52 (39.8% vs. 50%, *p* = 0.331). Additionally, both drugs are suggested to have comparable clinical response rates at week 8 (86.7% vs. 76.7%, *p* = 0.196) and at week 52 (72.3% vs. 76.7%, *p* = 0.642). Additionally, there were no significant differences regarding unwanted outcomes either (hospitalizations, steroid prescriptions, switching to a secondary anti-TNF agent, or the rates of an adverse event). Finally, CRP levels greater than 5 mg/L were correlated as a significant predictive factor for a poor disease outcome [87].

Regarding the pharmacokinetics, ADL, although being administered subcutaneously (s.c.), shares disposition similarities with IFX, i.e., a relatively low apparent volume of distribution, long elimination half-life, and relatively low systemic clearance (Table 4) [88,89,90]. Despite having many similarities with IFX, ADL has some pharmacological differences (Table 4 and Table 5).

The general goals of anti-TNF-α therapy in IBD can be summarized as follows: (i) inducing sustained endoscopic mucosal healing/endoscopic remission (as the primary endpoint), (ii) maintaining deep clinical remission (i.e., corticosteroid-free remission), (iii) preventing and reducing related complications of IBD disease, and (iv) improving the quality of life of IBD patients [91].

**Table 3:** Indications and “off-label” use of infliximab (IFX) and adalimumab (ADL) [78,92].

IFX	ADL
Crohn’s disease Ulcerative colitis Pediatric Crohn’s disease Pediatric ulcerative colitis Rheumatoid arthritis Ankylosing spondylitis Psoriatic arthritis Psoriasis	Crohn’s disease Ulcerative colitis Pediatric Crohn’s disease Rheumatoid arthritis Juvenile idiopathic arthritis Polyarticular juvenile idiopathic arthritis Active enthesitis-related arthritis Psoriatic arthritis Plaque psoriasis Pediatric plaque psoriasis Axial spondyloarthritis Hidradenitis suppurativa Uveitis Pediatric uveitis Panuveitis
Behcet’s disease Pyoderma gangrenosum Hidradenitis suppurativa Graft versus host disease Sjogren’s syndrome Uveitis Kawasaki disease	Behcet’s disease Pyoderma gangrenosum Alopecia areata Pemphigus Sarcoidosis Wegener’s granulomatosis

**Table 4:** Typical pharmacokinetic parameters after single dose of infliximab (IFX) [74,93] and adalimumab (ADL) [78,88,93] (in healthy subjects). * denotes the minimum post-induction C trough concentrations of patients with IBD suggested to be associated with an increased likelihood of mucosal healing at week 14 for IFX, and at week 4 for ADL [94].

Anti-TNF-α Agent	Dose	Route	Cmax µg/mL	Ctrough * µg/mL	Tmax Days	Clearance mL/h	Half-Life Days	Vd L	F %	AUC µg *h/mL
IFX	5 mg/kg	i.v.	126.2	>7	0.0875	11	14.1	4.8	100%	37,022
ADL	40 mg	s.c.	3.6	>7	7.9	16	14.5	7.9	64%	2167

i.v.—intravenous route; s.c.—subcutaneous route; Cmax—maximum plasma concentration; Tmax—time to reach maximum concentration; Vd—apparent volume of distribution; F—bioavailability; AUC—area under the curve.

**Table 5:** Differences in routes of administration and dosing of infliximab (IFX) and adalimumab (ADL) in CD and UC [78,92].

Biologics	Route	Induction Dose (CD and UC)	Maintenance Dose (CD and UC)
IFX	i.v.	5 mg/kg; Weeks: 0, 2, 6.	5–10 * mg/kg; Every 8 weeks.
ADL	s.c.	160 mg day 1, and 2; + 80 mg on day 15. or 80 mg Days: 1, 2, 15.	40 mg Every 2 weeks **. * Higher dose is recommended in *the case of * unsustained response to IFX ** Initial start on day 29

i.v.—intravenous route; s.c.—subcutaneous route; CD—Crohn’s disease; UC—ulcerative colitis.

## 6. Pharmacological Challenges of MAbs in the Example of Anti-TNF-α Agents IFX and ADL: Immunogenicity, Effectiveness, and Safety

One of the main challenges that MAbs are facing is the loss of response over time, leading to treatment failure. According to [95], around 20–30% of primary naïve patients with CD do not respond to induction therapy with anti-TNF treatments, which is referred to as the primary loss of response. Additionally, some IBD patients respond to the initial treatment, but are not able to achieve clinical remission, which is referred to as primary non-remission [96], and such patients are called partial responders [97]. In addition to this, 30–40% of IBD patients in remission on treatment become non-responders within one year of treatment, which is referred to as a secondary loss of response [95].

The main reason for the primary loss of response is an undesired immune reaction against the drug (MAb), i.e., immunogenicity. Immunogenicity implies the formation of anti-drug antibodies (ADAs), and affects the drug PK (increased clearance) as well as PD (effectiveness) [96,98,99]. Therefore, immunogenicity testing is a mandatory part of safety evaluation related to the approval of any biological product by regulatory agencies, such as the EMA or the FDA [100,101]. 

The etiology of treatment failure with MAbs is still not quite fully understood, which comes as no surprise due to the complex interplay of pharmacology, pathophysiology, and immunology [102]. Currently, despite some missing links, molecular assays for quantifying immunogenicity have become more sophisticated, and the knowledge on immunogenicity has been greatly extended, but there is still a need for improvement [103]. Under the current state-of-the-art methods, it was established that binding ADAs are categorized into two main categories with different ADA isotypes, which bind to different regions of a drug with various affinities. The first category of binding ADAs are non-neutralizing ADAs (non-NAb). They bind to the sites of the drug molecule, but without a direct pharmacological repercussion in situ; i.e., the drug’s pharmacodynamics are not affected at that moment. However, non-neutralizing ADAs have a notable impact on pharmacokinetics, as they increase the clearance of MAbs, which in turn has a direct effect on PD, as the drug exposure will likely be suboptimal. The second category of binding ADAs are neutralizing ADAs (NAb). They have a direct pharmacological impact due to their binding to an active drug site, which prevents drug–target binding. Hence, MAbs have a direct negative influence on therapeutic effectiveness [104,105]. Additionally, depending on the isotype of ADAs (e.g., IgM ADAs or IgG ADAs), individual immune responses may differ, which could have negative effects on disease progression or further development of neutralizing ADAs [106,107]. Hence, despite being initially detected by an assay, the real extent of ADAs regarding pharmacology cannot be immediately generalized without a proper detection assay [108]. Additionally, the incorrect terminology of non-neutralizing ADAs as “binding ADAs” (as both non-Nab and Nab are binding) results in misleading interpretations of immunogenicity assays. Consequently, comparing the results of immunogenicity and neutralization from various assays could be very misleading. Due to inconsistencies in reporting ADAs (underestimation and overestimation), some researchers propose the use of computational tools for pharmacokinetic modeling to distinguish the real clinical effects of ADAs on PK of MAbs [104,105].

For biologics in general, both types of ADA are likely clinically relevant, as both types of ADA can form immunogenic complexes and, in one way or another, decrease the therapeutic effectiveness of MAbs. Additionally, there is also a safety concern due to neutralizing ADA-mediated immunogenic complexes. However, in the case of anti-TNF-α agents, such complexes have not given rise to any safety concerns.

Researchers [95,96] also suggest that in addition to ADAs, other risk factors could explain the reasons for the primary loss of response. They include disease-related factors, such as localization, duration, and degree of inflammation, and inter-patient differences such as obesity, smoking, and hypoalbuminemia [104]. Additionally, there are differences in the genetic background of patients [104], as well as in the manufacturing process of biologics [102]. Hence, all these factors could influence PK and PD and, in the end, contribute to the unpredictability of a clinical outcome.

On the other hand, a secondary loss of response occurs mainly due to subtherapeutic concentrations of MAbs. This was observed in almost 70% of IBD patients, where, interestingly, ADAs were only detectable in roughly half of them [96,109].

IFX and ADL, in addition to their structural difference, differ in their dosing regimen and route of exposure (Table 5). This is very important for PKPD relationships, as well as immunogenicity. The bioavailability of IFX administered via the i.v. route is 100% and non-variable, while its biodistribution process is much faster compared to the s.c. route for ADL (Table 4) [110]. Clearly, as anti-TNF-α agents (and all biologics in general) are foreign proteins, immunogenicity is expected—especially if murine variable regions are present (e.g., in IFX). ADL, as the first fully human antibody, partially succeeded in overcoming the problem of immunogenicity and, within this context, has a better pharmacological profile. Nevertheless, for both drugs, the dose–exposure–response relationship needs to be improved [102,111].

Interestingly, a study from Brande et al. [112] suggested one additional reason for therapeutic failure in IBD patients; namely, an increase in the fecal loss of IFX was found to be related to “leaky gut”. It would be interesting to see additional research on this topic and determine its significance for the general PK of MAbs in IBD and beyond.

One may now ask the following question: which solutions might be proposed to respond to all the above-described challenges and improve the dose–exposure–response relationship of anti-TNF-α agents?

The first solution, at least to some extent, is to use biologics as an add-on treatment with one or more immunosuppressive agents, such as methotrexate, 6-mercaptopurine, or azathioprine [111,113]. Studies have confirmed that such combinations decrease the concentration of ADAs and, at the same time, increase the trough concentrations of IFX and ADL [111,113]. In this regard, suboptimal trough concentrations of MAbs seem to play an important role. A meta-analysis of MAbs concluded that concentrations above the IFX trough threshold of 2 μg/mL were more likely to be associated with the achievement of clinical remission and mucosal healing [114]. Similar findings were observed in a study with ADL, where UC patients in remission (remitters) had a mean ADL trough concentration of 10.8 μg/mL, compared to 6.18 μg/mL of non-remitters at week 52 [115].

The second solution, which is used by many clinicians, is therapeutic drug monitoring (TDM) [116]. The concept of TDM has various definitions [117,118,119], but its general goal is to achieve concentrations that are within a therapeutic window via dose titration [94,116]. Available guidelines of TDM in IBD exist; however, they are not sufficiently supported by high-quality data. Hence, more research is needed to gain a better understanding of how to achieve optimal clinical outcomes in IBD [120,121]. TDM can be divided into two categories [121,122]. The first is proactive TDM, where trough concentrations are determined and ADA measurements are performed in a defined time period in patients who start treatment with anti-TNF-α agents (induction), or in those who are undergoing a maintenance regimen. The overall goal of proactive TDM is to minimize disease progression and ADA development before non-responsiveness to MAbs occurs [121,123]. In the TAXIT trial, the proactive TDM of IFX in CD patients resulted in a decrease in relapses when the trough concentrations of IFX were 3–7 μg/mL (7% vs. 17%, *p* = 0.018) [123]. In another study, the proactive TDM of IFX in CD and UC patients was associated with higher rates of mucosal healing, lower rates of endoscopic inflammation, and fewer surgeries [124]. Similarly, the proactive TDM of ADL in CD patients was related to lower concentrations of ADAs, which in turn improved the clinical outcome [125].

The second category of TDM is reactive TDM, where trough concentrations are determined and ADA measurements are performed when there is a clinical recurrence of the disease, or when signs such as mucosal inflammation start to appear [120,121]. The reactive TDM approach is suggested by many associations within the field of gastroenterology [121], and has been shown to be a cost-effective strategy, compared to proactive TDM, which has been characterized as marginally cost-effective [120,121]. Interestingly, a stochastic simulated trial of CD patients on IFX showed that due to a decrease in the production costs of IFX, proactive TDM may be more cost-effective, which contradicts conventional thinking [122]. However, with reactive TDM, there is still a proportion of IBD patients who show subtherapeutic drug trough concentrations, with or without ADAs [121]. In a study by Papamichael et al. [126], it was shown that the proactive TDM of IFX in IBD patients following reactive TDM is associated with better clinical outcomes when compared to reactive TDM alone [126]. However, the results of a pragmatic trial by Bossuyt et al. [127] suggest that proactive TDM in IBD patients on IFX after a 1-year follow up has the same clinical outcomes as reactive TDM [127].

“Personalised anti-TNF therapy in Crohn’s disease study”—PANTS [128]—is the largest prospective study so far, which also comprised the TDM strategy. Suboptimal concentrations of IFX and ADL were found to be an independent factor associated with the primary non-response; namely, IFX and ADL concentrations (7 and 12 mg/L, respectively) at week 14 were associated with remission at week 54. Smoking was observed to be an independent factor related to IFX treatment failure, while obesity was considered to be an independent factor contributing to treatment failure with ADL. It is also suggested that obesity contributes to ADA development. Furthermore, dosing according to recommended regimens was characterized as “rarely helpful” in non-responders. This meant that only a small percentage of patients (0.12%) entered remission by week 54. At week 14, suboptimal concentrations of both drugs were associated with immunogenicity, i.e., higher ADA concentrations [128]. These findings again confirm the need for high-quality data and the improvement of personalized concentration-controlled dosing approaches in order to provide better optimization of the dose–exposure–response relationship for IFX and ADL in IBD patients.

It may be time to replace the concept of TDM, i.e., dose individualization based on a therapeutic window, and use more pharmacologically accurate interventions in order to achieve better success in the pharmacological outcome. “TDM is dead. Long live TCI!”, by Holford et al. [129], provides a clear rationale of abandoning TDM, and stresses the benefits of using an alternative approach: target concentration intervention (TCI). In fact, TCI is pharmacologically driven as it implies PKPD concepts for predicting individual parameters, which are used for suitable dose calculation, instead of just empirical guidance based on a therapeutic window or minimal trough concentration as used in TDM. In other words, by using a TCI approach, we can aim for a target effect associated with a target concentration, rather than hoping for a beneficial outcome based on a minimal trough concentration. The final goal of TCI is a maintenance dose (predictable from PK) and corresponding dosing interval that achieves steady-state target exposure in patients [129,130]. However, despite being pharmacologically accurate, TCI is still not sufficiently acknowledged by the majority of clinicians.

The third solution could be the use of computational methods and tools that are under the umbrella of clinical pharmacology and pharmacometrics and expanding towards the quantitative systems pharmacology (QSP) area. In other words, bridging pharmacokinetic and pharmacodynamic modeling (PK/PD modeling) with more mechanistic approaches, such as physiologically based pharmacokinetic modeling (PBPK) and QSP, is expected to advance the field of personalized (and precision) medicine. The rationale behind this lies in the context of ADA formation; inter-patient variability, i.e., between-subject variability; and the interplay of pathophysiology and immunology [131,132], so the suggested dosing regimens (and drug concentrations) will not be the same for all patients. By employing, for example, population PKPD modeling, we can discover sources of variability in the target population, identify significant covariates (such as obesity), and contribute to better treatment effectiveness [17]. PBPK/PD modeling, on the other hand, offers the integration of both physiology and anatomy (organs and tissues connected by blood flow rates) with physicochemical drug-related parameters that impact ADME [133].

PBPK modeling was recently utilized within population approaches to explore the pharmacokinetics of IFX in pediatric IBD patients [134]; namely, the PBPK model showed that only half of the children reached an optimal trough IFX concentration when the dosage was in accordance with the standard regimen [134]. It is suggested that either higher doses of IFX or changing the dosing interval is needed for better effectiveness in pediatric patients. Similarly, due to the growing knowledge on QSP and state-of-the-art computational methods, it is expected that the success of pharmacological outcomes, as well as disease progression, could be easily predicted on a case-by-case basis. When considered from the perspective of clinicians, QSP is still in its infancy. However, a recently published example of a QSP model of IBD proves how this field of pharmacology is rapidly expanding [135].

In addition to the factors that influence the effectiveness of biologics, i.e., anti-TNF-α agents, an equally important term is safety. IFX and ADL are well-tolerated overall [136]. However, there are still safety concerns, which are evident in the literature. The reported acute infusion reactions associated with IFX are believed to be due to ADA. Reactions consist of fever, chills, dyspnea, and headaches. Delayed reactions (3–12 days after infusion) include myalgia, arthralgia, urticaria, lip edemas, and pruritus. Additionally, cases of serum sickness-like reactions have also been reported [92,137].

In a long-term study of IFX effectiveness in UC, almost 30% of patients discontinued IFX infusion due to adverse events [138]. Other studies report an increased risk of serious infections; a likely increased risk of malignancies (possibly due to combination with immunosuppressive agents); and immune-related complications, such as drug-induced lupus, demyelination, neurologic reactions, and psoriatic-like lesions [139,140,141]. The occurrence of antinuclear antibodies (ANA) and the induction of lupus have also been linked to anti-TNF-α agents, including IFX and ADA [140,141]. However, lupus symptoms are not life-threatening and have been shown to be resolved after discontinuation of the drug. Paradoxically, anti-TNF-α agents have been successfully used to treat lupus, so the exact mechanism of this reaction, for now, remains unknown. In a recent study [142], ANA development was suggested to be a risk factor for ADA development, for both IFX and ADL, but in patients with RA. To the best of our knowledge, a similar study for IBD patients has not been performed.

Treatment with IFX has been linked to the reactivation of tuberculosis, HBV infection, anaphylaxis, hepatotoxicity, hematologic toxicity, and adverse outcomes in patients with heart failure [139]. As there are still many unanswered questions, and causal relationships with some adverse reactions have not been confirmed, or data are missing, further studies on IFX safety are needed. Prior to starting IFX therapy, a thorough assessment of a patient’s history is mandatory. Contraindications include active infections, latent tuberculosis, moderate to severe heart failure, and a history of multiple sclerosis. All patients should also be inspected for their vaccination status, as some vaccines are contraindicated. Annual screening for some diseases (cancer, tuberculosis, and HBV) is highly encouraged for high-risk patients [139,143]. 

Regarding ADL, due to its similarity to IFX, the same safety concerns (in different indications) are reported [144,145]. Local injection site reactions from ADL were less common [146] compared to IFX, but surprisingly, the overall rates of adverse events with ADL for some groups of patients were less favorable; namely, in psoriatic patients, the rate of serious adverse events per 100 patients was 7–9 for ADL vs. 4–8 for IFX [145]. However, approvals of new indications and the availability of new data have not reported new safety signals [144]. In an intuitive sense, it can be also stated that patients who show signs of any adverse reaction with IFX could, in theory, show the same reaction when IFX is exchanged for ADL.

The use of anti-TNF-α agents, IFX and ADL, during pregnancy was not related to any adverse pregnancy outcomes, nor were there increased risks of infant infections. Due to in utero exposure and increased risks of infections, all live vaccines (e.g., Bacillus Calmette–Guerin vaccine: BCG, or rotavirus vaccine) should be avoided for 12 months after birth [147]. Additionally, infants exposed to IFX via breast milk should not receive any live vaccines unless the concentrations of MAbs are undetectable [148,149]. However, the majority of the literature data and expert group opinions prior to March 2022 suggest how the use of IFX and ADL is “compatible with breastfeeding” [147,150]. A mother could easily, unaware of the danger of prior in utero exposure of an infant, incorrectly think that due to “compatibility with breastfeeding”, it is safe to administer the vaccine to an infant. Hence, we advise consulting the relevant up-to-date literature, and stress the importance of proper physician–patient communication in order to prevent cases of infant death [151].

In conclusion, both IFX and ADL can be considered as equally safe and comparable regarding clinical outcomes in adult IBD patients (naïve to anti-TNF therapy). However, despite its long-term use in IBD patients, their dose–exposure–response relationship must still be improved [86,98,152]. Hence, the immunogenicity, effectiveness, and safety of MAbs, i.e., anti-TNF-α agents, will remain a topic of utmost importance.

## 7. Misconceptions in the Era of Biosimilars

The EMA pioneered the approval and regulatory process of biosimilars by introducing the first biosimilar on the EU market in 2006, which was Omnitrope^®^ [153], a recombinant human growth hormone (somatotropin), while Zarxio^®^ (filgrastim) was the first biosimilar ever approved by the FDA in 2015 [154]. According to some projections, the global biosimilar market will rapidly expand in the next few years, whereas MAbs will continue to be among the leading segments. The fastest-growing market is Asia-Pacific, while the largest market is North America. It is expected that the global market will grow from EUR 14.8 billion in 2021 to EUR 42.5 billion by 2026 [155]. The major advantage of biosimilars is their cost-effectiveness due to their lower research and development costs, compared to originators. In addition, production issues, along with the very competitive milieu for manufacturers and patent battles, are still relatively challenging. For example, by 2023, more than 15 patents of oncology biologics will expire [4,155]. However, establishing biosimilars as an alternative of equal worth to reference biological medicine (or originator) is accompanied by some misconceptions and controversy.

The EMA defines a biosimilar as a “biological medicine highly similar to another already approved biological medicine in the European Union (EU) for which marketing exclusivity rights have expired” [156]. Manufacturers of biosimilars must demonstrate through comparability studies (in three steps), based on “the totality of evidence” (a phrase coined by the FDA), that biosimilars and reference biological medicines (or originators) are “highly similar, notwithstanding natural variability inherent to all biological medicines”, and that there are “no clinically meaningful differences” in “quality, safety, and efficacy” between them. In other words, the EMA (as well as the FDA) demands a head-to-head comparison of two products in a step-wise approach that is individually tailored for every biosimilar. This is implied in step one: analytical (i.e., the characterization of physical and chemical properties, as well as purity) and functional comparative studies (i.e., the characterization of biological activity and immunochemical properties); step two: comparative non-clinical studies, i.e., in vitro and possible in vivo studies, aiming for a pharmacodynamic and toxicological comparison; and step 3: comparative clinical studies, i.e., pharmacokinetic and pharmacodynamic comparison, as well as the comparison of effectiveness, safety, and immunogenicity if needed [153].

The FDA’s definition of a biosimilar [157] is similar to that of the EMA: “A biosimilar is a biological product that is highly similar to and has no clinically meaningful differences from an existing FDA-approved reference product”. The FDA has four subcategories for the regulatory assessment of biosimilarity of a biosimilar: not similar, similar, highly similar, and fingerprint-like similar. However, only the last two categories satisfy the criterion for having a “true” biosimilar title. In the US, biologics are licensed and referred to as drugs or biologics, while in the EU, they are authorized, and referred to as biologic medicines [158,159].

One must also bear in mind that the FDA and EMA use different vocabulary/terminology, which could lead to confusion. The main differences are explained by S. Niazi [158,159], who points out that “no clinically meaningful differences”, for the FDA, actually means “establishing safety, purity, and potency”. “Safety” (defined by the FDA) is referred to as immunogenicity (for the EMA), while “purity” (defined by the FDA) is referred to the characterization of physical and chemical properties (for the EMA). “Potency” (i.e., safety and effectiveness) (defined by the FDA) is referred to as functional comparative in vitro/in vivo studies (or human trials) (for the EMA), due to the fact that “minor differences in clinically inactive components” are acceptable for the FDA, i.e., variability in the manufacturing processes is expected. The terms that should also be distinguished and should not be used interchangeably, as S. Niazi highlights, are: (i) comparability (meaning a comparison) vs. similarity (meaning sameness); (ii) efficacy (meaning the degree of clinical response–maximal response = Emax) vs. effectiveness (meaning the extent of achieving the intended effect, or the result of the comparison of two clinical responses); and (iii) an innovator drug (meaning a newly invented therapy) vs. an originator (meaning therapy that has its own biological origin) [158,160].

When discussing biosimilars, it is important to stress how they are not generics, as is often indicated [161]. While generics are identical to an originator (i.e., referent drug) in terms of active pharmaceutical ingredients, biosimilars are only “highly similar”. Moreover, the chemical synthesis of generics is more or less identical, while for biosimilars, it is well-established that they have inherent variations due to complex manufacturing processes (i.e., microheterogeneity and post-translational modifications). Hence, biosimilars cannot be identical, and even if they originate from the same manufacturer, there is still some batch-to-batch variability. Of course, all batches of both biosimilars and originators must always show consistency within very narrow limits. In other words, regulatory agencies (the EMA and the FDA) demand the identification of critical quality attributes (CQAs) among the comparability of quality attributes (QAs), which aims to provide evidence of the “high similarity” of biosimilars, on which biosimilar approval relies [162,163,164,165].

Biosimilars are referred to by a variety of names in the literature, e.g., biosimilar products, off-patent biopharmaceuticals, subsequent entry biologics, follow-on biologics, and me-too biologics. The last two terms are sometimes used to refer to biobetters (also known as biosuperiors or me-betters). Biobetters can be considered as a newly designed modality (new molecular entry) that originated from an existing biologic [166,167].

One may ask the following question: when does a biosimilar actually become a biobetter? Although the regulatory definition is lacking, the “transition” can be considered only when improvements in manufacturing, biopharmaceutical, and/or pharmacological properties are demonstrated. However, a biobetter must maintain the same pharmacological purpose. This new molecular entry (FDA term), i.e., the biobetter, could have better effectiveness, higher selectivity, a better safety profile (i.e., decreased immunogenicity), better bioavailability, or improved stability [168]. The great advantage of biobetters, compared to biosimilars, is that patents are not obstacles. However, on the other hand, an exhaustive regulatory process takes a much longer time, so the cost-effectiveness of biobetters is higher. Finally, the probability of the technical and regulatory success of a biobetter is much lower compared to a biosimilar (41% vs. 65%) [166]. Interestingly, the first anti-TNF-α biobetter to enter the global market was in fact ADL—a biobetter of IFX. IFX was developed by Janssen under the trade name Remicade^®^. However, as its rate of immunogenicity was high, Abbot developed a biobetter, which was registered under the trade name Humira^®^ [169]. Anecdotally, the term “biobetter” was introduced a few years after that (in 2007) at a conference on biologics in India [170].

From the literature, it is obvious that among some scientists and physicians, biosimilars (and biobetters) are welcomed with less enthusiasm [171,172,173]. For some, the only benefit of biosimilars is their reduced cost, while other benefits are imbued with suspicion. Thus, it is important to address misconceptions [174] related to biosimilars. The most common misconception is that biosimilars are less safe and less effective compared to the original biologic product. However, this is simply not true. Biosimilars undergo very rigorous and extensive testing. In fact, the regulatory approval of biosimilars is more voluminous compared to that of generic drugs. At the same time, manufacturers are dedicated to providing safe and effective biosimilars. Overall, biosimilars have been used in Europe for more than 15 years now, which is a relatively long time to recognize any potential dangers associated with their use [175].

Some researchers may question the safety of biosimilars by referring to an epidemic of pure red-cell aplasia in Thailand, where, due to leachate from rubber, toxicity occurred [176]. Additionally, recently, some rumors about cases of death related to biosimilars have also spread [177]. However, leachate from the rubber was not a product of the direct toxicity of a biosimilar per se, while Lyman et al. [177] described these rumors as an exaggeration.

Other researchers point out how biosimilar approval data (non-clinical and clinical) are different compared to the data needed for the approval of an originator [178]. This implies the existence of safety concerns related to the approval of biosimilars. However, in order to be approved in the EU, a biosimilar relies on the data from the originator, and as a part of demonstrating biosimilarity, a biosimilar must undergo extensive comparability studies, which prove its comparable (similar) physicochemical properties, biological activity, safety, and effectiveness [175,179]. One benefit of this is that there is no need to repeat the entire clinical development program of an originator.

Another question that often increases pre-existing suspicions about biosimilars is “How similar is similar enough?”. “Similar” means just that—only similar—and not “the same”, i.e., not identical [178]. At first glance, the phraseology behind these words (“similar”, i.e., not “identical”) without a scientific context may sound different. However, in the regulatory context, it is not. As previously mentioned, it is not even expected that biosimilars are the same, i.e., identical, because it is well-established that they are not, and neither are the original versions from the same manufacturer [179,180]. In fact, “similar” refers to the satisfaction of all levels of regulatory requirements in a biosimilar comparability exercise, in addition to approval, which is based on the “totality of evidence”. This means, in the regulatory context, that “similar” is just as good as “identical”.

## 8. Controversies in the Era of Biosimilars: Interchangeability

In the EU, small-molecule generics have the same quantitative and qualitative composition of the active substance, the same dose, and the same pharmaceutical form as the reference product [181,182]. Appropriate bioavailability studies need to demonstrate comparable relative bioavailability between a generic and a reference product. However, small-molecule generics do not need to be identical to the reference product (similar to biosimilars not needing to be identical), as differences in salt form, excipients, particle size, etc. are still allowed. If bioequivalence is confirmed (usually in healthy volunteers), small-molecule generics are considered therapeutically equivalent to the reference product. It can be assumed that such small-molecule generics are also interchangeable with the reference product [182,183]. Hence, interchangeability, within the EU, can simply be considered as the possibility of exchanging one medicine for another medicine under the condition that they produce the same clinical effect [175,184]. The term “medicine” here includes small-drug molecules or biologics, as well as generics or biosimilars. However, not all generics with the same active substance will be interchangeable due to the fact that the concept of substitution is based on immediate release formulations. In some cases, the same active pharmaceutical ingredient classified as a small-molecule drug (e.g., doxorubicin or tacrolimus) cannot be considered interchangeable due to differences in formulation (e.g., aqueous solution vs. pegylated liposomal solution of doxorubicin, or retard vs. immediate-release formulations of tacrolimus) [182].

The term interchangeability includes switching and substitution as a part of exchange practices in the EU [175]. If the exchange is carried out based on the decision of an EU physician (prescriber), it is called switching. However, if the same exchange is carried out at the pharmacy level by an EU pharmacist, i.e., without consulting the prescriber, it is called substitution [175,185].

There are two types of switching: non-medical and medical. Non-medical switching, in contrast to medical switching, is not related to any of the safety, effectiveness, or adherence concerns of a particular medicine [186]. In other words, there is no medical reason that could influence the prescriber’s decision [187]. It should also be pointed out that “therapeutic substitution”, which is replacing one therapeutic agent with another that is chemically different, should not be confused with the term “substitution”, which is defined under the term interchangeability [182].

In most countries, physicians are the ones who make the decision to switch from an originator to a biosimilar, or a biosimilar to another biosimilar (with the patient’s consent). For example, in the United Kingdom, switching from an originator IFX (Remicade^®^) to its biosimilars (Inflectra^®^ and Remsima^®^) is a widespread practice [188]. On the other hand, switching was not recommended in Spain until recently, which is explained by the fact that the cost of treatment with an originator is similar to the cost of that with a biosimilar. However, according to a more recent source [189], this was refuted, and Spain is said to be also on the way to making its health care system more sustainable.

Within some EU countries and in the USA, pharmacists can substitute conventional medicines with an equivalent generic drug [190]. For biosimilars, the situation is different, and biosimilar substitution in the EU was more or less an exception rather than the rule [191]. Recently, this has started to change, and substitution is starting to be encouraged [188]. For example, in France, substitution is currently allowed for treatment-naïve patients and when an originator and biosimilar are from the same biologic group, defined by a reference biologic and its corresponding biosimilars listed by brand name. However, this substitution can only be applied if approved by a physician [192]. The Czech Republic was the first country that permitted biosimilar substitution at the pharmacy level in 2020, while in Germany, the automatic substitution of biosimilars is supposed to be introduced this year (2022) [188].

In addition to the previously mentioned misconceptions that biosimilars are facing, the concept of biosimilar interchangeability can be considered as controversial. The reason for this is that scientific evidence regarding the effectiveness and safety of interchangeability, i.e., switching and substitution, is still lacking (in general) [193]. On the other hand, the first-ever randomized trial of switchability, called NOR-SWITCH, showed that the switching of the IFX originator to its biosimilar (CT-P13) in patients with CD, UC, psoriatic arthritis, chronic plaque psoriasis, rheumatoid arthritis, and spondylarthritis did not result in any different outcomes when compared to continuous therapy with the originator [194]. Moreover, the frequency of adverse effects was comparable, while the NOR-SWITCH extension study (2019) concluded that the switching of the IFX originator to its biosimilar CT-P13 is safe and effective [195]. Similarly, results from the PLANETRA trial showed that the switching of the IFX originator to a biosimilar (CT-P13) in RA patients had comparable results in terms of safety, effectiveness, immunogenicity, PK, and PD [196].

Regarding the effect of switching to biosimilars of ADL, the data are not comprehensive, as with IFX; however, initial studies show comparable results of switching to biosimilars ABP501 and MSB11022 in terms of clinical similarity, safety, effectiveness, and immunogenicity for chronic plaque psoriasis [197,198].

In IBD patients, ADL biosimilars ABP-501 and SB5 were also found to be comparable to the originator in terms of safety and effectiveness [199]. The results of other studies [200,201] of switching to ADL biosimilar SB5 were consistent with previous findings.

The analysis of the European Public Assessment Reports (EPAR) and pharmacovigilance safety surveillance reports (up to August 2020), regarding the short- and long-term safety and interchangeability of approved biosimilars, including three IFX biosimilars and six ADL biosimilars, among others, concluded that a single and multiple switching between the originator and biosimilar did not have any negative effects on safety, effectiveness, or immunogenicity [202].

Positive expectations of clinicians about the effectiveness and safety of medicines that they are prescribing contribute to better patient–physician relationships. Hence, their subjective opinion can impact a patient’s satisfaction with a particular medicine and enhance compliance [203]. Interestingly, clinicians with different specialties have different perceptions about biosimilars. Among EU clinicians, rheumatologists showed higher levels of skepticism, compared to gastroenterologists, in terms of trustworthiness of biosimilars, as well as their effectiveness and safety [204].

In another survey from 2016 [205], among the 1201 US clinicians who prescribed biologics, only 44.8% agreed with the statement that biosimilars are safe and appropriate for use in naïve and existing patients. These results imply that the majority of physicians believed that switching to biosimilars could be a safety risk. Additionally, a sizeable minority did not even know what medicines within their specialty were biologics, or if they were approved as biosimilars in the US. However, 76% responded that they would like to learn more about the safety and effectiveness of biosimilars [205]. It would be interesting to analyze the results of this survey today among the same clinicians.

The interchangeability of biosimilars is also related to a phenomenon of nocebo response (or nocebo effect) [206]. Nocebo has a negative influence on treatment effectiveness due to the patient’s expectations and beliefs, but this negative influence is totally unrelated to the pharmacological effect of a biosimilar [207]. For example, in one study [208], patients with RA who switched from the originator to a biosimilar of IFX showed a decreased retention rate after switching. Additionally, some patients asked to be switched back to the originator despite not showing any signs of disease activity [208]. In the same study, when these patients were excluded, there was no difference in the retention rate. Similarly, in another study [209], patients chose to discontinue treatment with the biosimilar of IFX, despite not having any disease activity. The authors also suggested that subjective reasons, i.e., negative expectations, could be a factor that negatively contributes to the successful treatment of RA patients with the IFX biosimilar.

Decisions regarding the interchangeability of generics and biosimilars of EMA are made by national regulatory authorities due to different clinical practices within EU countries [210]. On the other hand, the FDA issued its final guidelines on biosimilar interchangeability in 2019 [211]. Thus, by not having a clear official standpoint of interchangeability, the EMA is contributing further to existing misconceptions and controversies about biosimilars. Currently, clinical evidence supporting safety concerns related to IFX biosimilars and interchangeability in IBD patients is lacking. Hence, switching from IFX originators to IFX biosimilars is considered to be safe, with comparable effectiveness. The switching of ADL showed encouraging data; however, additional studies on bigger cohorts are still needed to confirm the safety and effectiveness of ADL biosimilars and their interchangeability in IBD patients. Regarding other biosimilars on the global market and their corresponding indications, additional trials and extensive post-marketing surveillance data are needed.

## 9. Conclusions

The incidence of chronic inflammatory disorders, such as IBD, is increasing worldwide. Despite the domination of MAbs (and biosimilars) in treating such diseases, therapeutic goals are not achieved in all patients. Relapsing and remitting courses of the disease, the complex interplay of physiology and immunology, and PKPD variability among patients summarize the reasons that therapeutic outcomes are not successfully achieved in all patients. The major challenges of anti-TNF-α agents (and MAbs in general) are still related to immunogenicity, effectiveness, and safety. Hence, the gaps in the current knowledge regarding their PKPD must be addressed to improve their dose–exposure–response relationship.

Critical tasks include discovering multiple disease factors and underlying molecular mechanisms, revealing the causes of variable disposition mechanisms of MAbs, and a search for better bioanalytical evaluation approaches. Due to constant changes within this complex field, guidelines for the management of IBD should also be frequently updated.

The global use of biosimilars is suggested as a key strategy towards sustainable health care. Despite common misconceptions (effectiveness and safety) and controversy (interchangeability), biosimilars have the power to ease the burden of many diseases, including IBD.

The scientific literature and regulatory demands in the EU are constantly evolving, but the need for trustworthy information is still prevalent. Hence, pharmacovigilance activities are of great use if safety signals are properly detected and carefully monitored.

## 10. General Limitations and Prospects

Despite the fact that MAbs are a game changer in treating chronic inflammatory diseases, more research is needed for a better understanding of inflammation and signaling cascades, in order to successfully modulate the immune response.

Existing clinical findings should be supplemented by clinical trials (from patients) with a longer trial duration, observational studies, and real-world evidence (RWE) data. Additionally, it is expected that pharmacometrics approaches will be more widely utilized as a powerful tool for advancing precision medicine.

Regulatory agencies are expected to provide better harmonization across EU countries, and be more involved in implementing guidelines with respect to biosimilar-based treatments. In addition to proper terminology use, interchangeability and switchability, it is expected that the topic of indication extrapolation will also be more widely acknowledged in the future.

From the clinical point of view, suggested approaches, such as TCI, novel predictive biomarkers, and novelties in the area of pharmacogenomics, are expected to contribute to better individualized treatments.

As the large biologic market is expecting further expansion, it is also important to stay up to date with new information and find an adequate way of delivering it to health care specialists. Advancements in manufacturing technologies for MAb-based therapeutics are also expected.

Finally, with recent developments, new emerging biotherapeutics, such as antibody–drug conjugates and bispecific antibodies, are slowly but surely writing the next chapter in the pharmacology of antibody-based therapeutics. Hence, a pharmacological armamentarium against many chronic diseases, including IBD, will be further expanded.

## Figures and Tables

**Table 1:** General differences in small-molecule drugs vs. biologics [8,9].

Small-Molecule Drugs	Biologics
Low molecular weight (<0.5 kDa)	High molecular weight (>2–5 kDa)
Small size + lipophilicity allows passage across barriers	Due to its large size, penetration is not expected across barriers
Homogenous mixtures	Heterogeneous mixtures, with possible variants
Well-defined structure	Structure may not be known (or not well-defined)
Physicochemically less complex	Physicochemically very complex
Easily synthesized	Made from live cells and organisms
Less critical steps in the manufacturing process	Many critical steps in the manufacturing process
Very well characterized (methodology is known)	Not easily characterized
Stable; heat stable	Not stable; heat sensitive
Administered orally	Usually administered parenterally (intravenously, intramuscularly)
Relatively short half-life; daily dosing regimen	Longer half-life (days to weeks); monthly dosing regimen
High risk for “off-target effects”	High selectivity and specificity for a target
Metabolism by liver enzymes—Cytochrome P450 (CYP)	Catabolism (degradation) and limited toxicity
Higher risk of drug interactions and toxicity due to CYP	Drug interactions are less common
Immunogenicity is not expected	Immunogenicity is a big challenge
Treatment is not expensive, i.e., lower costs of development	Treatment is very expensive, i.e., development costs are much higher
Longer development cycle	Shorter development cycle
Well-defined mechanisms of action	Pleiotropism in pharmacological effects
Rigid in terms of structure manipulation	Structure manipulation is possible and can offer an enhancement of pharmacological properties

**Table 2:** Common symptoms and extraintestinal manifestations of IBD [38,39,40].

Symptoms of IBD	Extraintestinal Manifestations of IBD
Fever	Arthritis
Fatigue	Ankylosing spondylitis
Diarrhea	Osteoarthropathy
Blood in stool	Osteoporosis
Abdominal pain	Erythema nodosum
Abdominal discomfort	Pyoderma gangrenosum
Nausea, Vomiting	Stomatitis
Weight loss	Drug rashes
Cramping	Brittle nails
Loss of appetite	Hair loss
Mouth sores	Primary sclerosing cholangitis
Rectal pain	Bile-duct carcinoma
Fail to defecate	Pancreatitis
	Colorectal cancer
	Fatty liver
	Portal fibrosis
	Autoimmune hepatitis
	Gallstones
	Uveitis, episcleritis, retinal diseases, dry eyes
	Anemia
	Thromboembolism
	Depression, anxiety
